# Pegunigalsidase alfa: a novel, pegylated recombinant alpha-galactosidase enzyme for the treatment of Fabry disease

**DOI:** 10.3389/fgene.2024.1395287

**Published:** 2024-04-12

**Authors:** Dominique P. Germain, Ales Linhart

**Affiliations:** ^1^ Division of Medical Genetics, University of Versailles–St Quentin en Yvelines (UVSQ), Paris–Saclay University, Montigny, France; ^2^ Second Department of Medicine, Charles University, General University Hospital, Prague, Czechia

**Keywords:** Fabry disease, PEGylation, agalsidase, non-inferiority trial, renal function

## Abstract

Fabry disease, a rare X-linked genetic disorder, results from pathogenic variants in *GLA*, leading to deficient lysosomal α-galactosidase A enzyme activity and multi-organ manifestations. Since 2001, enzyme replacement therapy (ERT), using agalsidase alfa or agalsidase beta, has been the mainstay treatment, albeit with limitations such as rapid clearance and immunogenicity. Pegunigalsidase alfa, a novel PEGylated recombinant alpha-galactosidase, offers promise as an alternative. Produced in plant cells, pegunigalsidase alfa exhibits enhanced stability, prolonged half-life, and reduced immunogenicity due to pegylation. A phase 1/2 clinical trial demonstrated Gb3 clearance from renal capillary endothelial cells and its 48-month extension study revealed notable outcomes in renal function preservation. Three phase 3 clinical trials (BRIDGE, BRIGHT, and BALANCE) have shown favorable efficacy and safety profile, although caution is warranted in interpreting the results of BRIDGE and BRIGHT which lacked control groups. In BALANCE, the pivotal phase 3 trial comparing pegunigalsidase alfa with agalsidase beta, an intention-to-treat analysis of the eGFR decline over 2 years showed that the intergroup difference [95%confidence interval] in the median slope was −0.36 mL/min/1.73 m^2^/year [−2.44; 1.73]. The confidence interval had a lower limit above the prespecified value of −3 mL/min/1.73 m^2^/year and included zero. Despite challenges such as occasional hypersensitivity reactions and immune-complex-mediated glomerulonephritis, pegunigalsidase alfa approval by the European Medicines Agency and the Food and Drug Administration represents a significant addition to Fabry disease therapeutic landscape providing an option for patients in whom enzyme replacement therapy with current formulations is poorly tolerated or poorly effective.

## Introduction

**FIGURE 1:**
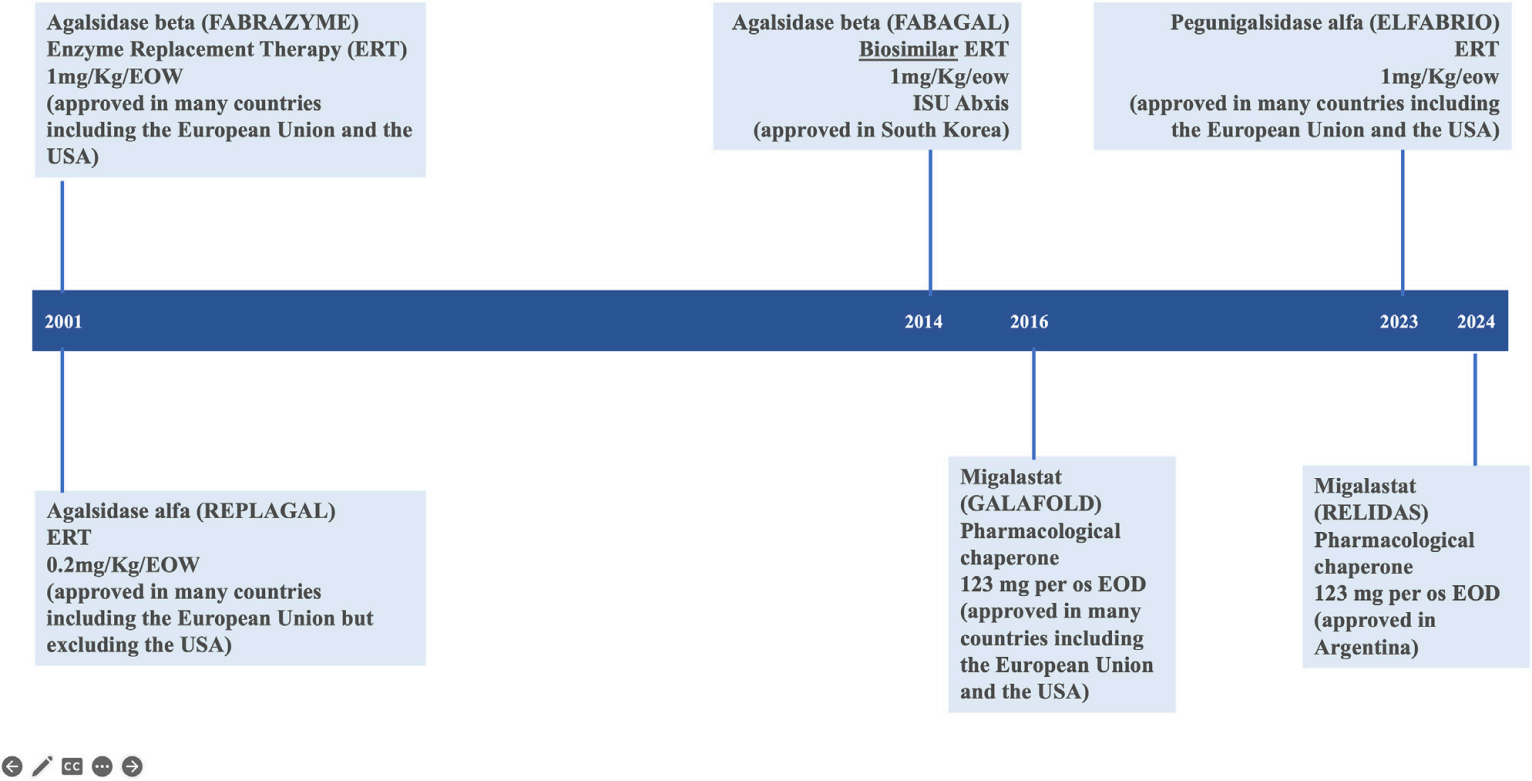
Current therapeutic landscape in Fabry disease.

Fabry disease (FD; Online Mendelian Inheritance in Man^®^ OMIM #301500) is an X-linked, genetic disease due to pathogenic variants in the *GLA* gene (OMIM #300644; HUGO Gene Nomenclature Committee ID: 4296; NCBI reference sequence: NM_000169.3) (Germain et al., 1996; Germain et al., 2020) encoding the lysosomal α-galactosidase enzyme (α-GAL, UniProt ID: P06280) resulting in its absent or markedly decreased activity in lysosomes. Multiple organ systems are affected (Tuttolomondo et al., 2013; Ortiz et al., 2018; Wanner et al., 2023) with a wide spectrum of progressive clinical phenotypes (Germain, 2010; Lin et al., 2015; Liu et al., 2015; Namdar, 2016; Linhart et al., 2020; Pieroni et al., 2021; Vujkovac et al., 2021; Levstek et al., 2023), particularly among female patients (Echevarria et al., 2016). Since 2001, enzyme replacement therapy (ERT) with exogenous human α-galactosidase has been the mainstay of FD-specific treatment to stabilize, delay or prevent progressive organ damage and improve disease symptoms (Germain et al., 2022). There are two preparations of ERT available in most countries: agalsidase alfa (Replagal^®^, Takeda) (European Medicines Agency, 2024d) and agalsidase beta (Fabrazyme^®^, Sanofi) (European Medicines Agency, 2024b). A chaperone therapy, migalastat (Galafold^®^, Amicus) (Germain et al., 2016; European Medicines Agency, 2024c), was approved for the treatment of patients with *GLA* amenable variants in the European Union in 2016 and in the United States in 2018 (Figure 1). The accumulation of clinical trial and real world evidence over the last 20 years has shown that enzyme replacement therapy (ERT) via the lifelong intravenous infusions of gene activated agalsidase alfa (Schiffmann et al., 2001) or recombinant agalsidase beta (Eng et al., 2001) every other week is safe and clinically and biologically effective in patients with FD (Wanner et al., 2018; Spada et al., 2019; Germain et al., 2022; Burlina et al., 2023; Wanner et al., 2023). Enzyme replacement therapy counters the lack of functional α-galactosidase, which (in the absence of treatment) leads to the progressive accumulation of globotriaosylceramide (Gb_3_) and globotriaosylsphingosine (lyso-Gb_3_) in tissues, plasma, and urine. The enzyme preparations agalsidase alfa (Replagal^®^, Takeda) and agalsidase beta (Fabrazyme^®^, Sanofi) have identical amino acid sequence, but notably differ with regard to their biotechnological production processes affecting their glycosylation and dosing (0.2 mg/kg *versus* 1.0 mg/kg every other week for agalsidase alfa and agalsidase beta, respectively) (Lee et al., 2003). Upon the initiation of enzyme replacement therapy, Gb_3_ levels in the plasma, kidney, heart, skin and liver and lyso-Gb_3_ levels in the plasma typically fall in a dose-dependent manner to near-normal values (Eng et al., 2001; Schiffmann et al., 2001; Tøndel et al., 2022; Burlina et al., 2023). Despite treatment advances with ERTs, limitations exist. Some patients show limited improvement with FD treatment (Lidove et al., 2010) and even optimally treated patients with classic FD may occasionally develop albuminuria, proteinuria, and decline their glomerular filtration rate in some cases to end-stage chronic kidney disease prompting renal replacement therapy. The effectiveness of enzyme replacement therapy in Fabry disease is notably limited by the rapid clearance of the current enzymatic agalsidase preparations from the circulation, which limits distribution of the therapeutic to the many tissues and organs affected by FD. Furthermore, frequent development of antidrug antibodies (Lenders and Brand, 2018) some of which with neutralizing capabilities can hamper clinical efficacy (Wilcox et al., 2012; Lenders and Brand, 2018). Infusion-associated reactions (IARs) may affect treatment compliance (Azevedo et al., 2021).

### The preclinical and clinical development of a PEGylated agalsidase

The need for new treatment modalities prompted the development by Protalix Biotherapeutics of PRX-102 or pegunigalsidase alfa, a novel PEGylated agalsidase alfa (Ruderfer et al., 2018) that was recently approved for the treatment of FD in the EU and US by the European Medicines Agency (EMA) and the Food and Drug Administration (FDA), respectively. The recombinant protein moiety of pegunigalsidase alfa is produced in plant cells (Kizhner et al., 2015; Tekoah et al., 2015); this contrasts with the methods used to produce agalsidase alfa (HT-1080 human fibrosarcoma cell line) and agalsidase beta (Chinese hamster ovary cell line). The plant derived enzyme production may be more resistant to potential contamination and biological culture infection by adventitious agents such as vesivirus 2117, which severely compromised agalsidase beta production in 2009–2011 (Linthorst et al., 2011; Smid et al., 2011).

Pegunigalsidase alfa is a hydrolytic lysosomal neutral glycosphingolipid specific enzyme. It is a PEGylated and cross-linked, chemically modified recombinant alpha-galactosidase that is produced by genetically-modified *Nicotiana tabacum* plant cells. The aminoacid sequence of one subunit of pegunigalsidase alfa consists of 405 amino acids, of which 398 amino acids are identical to human alpha-galactosidase A with six additional amino acids included at the C-terminal to encode an endoplasmic retrieval signal, and an additional glycine at the N-terminus derived from the signal peptide (Food and Drug Administration (FDA), 2023).

As compared to mammalian cell line production, the plant-derived glycosylation is less complex and does not contain mannose-6-phosphate moieties (M6P), requiring another mechanism of cellular uptake than M6P receptors (Prabakaran et al., 2011; Marchesan et al., 2012; Kizhner et al., 2015; Shen et al., 2016; Tian et al., 2019).

Pegunigalsidase alfa is modified with 2 kDa polyethylene glycol (PEG) moieties, producing PEGylated protein subunits cross-linked and covalently bound into homodimers. Additional PEG moieties are also attached to surface lysine residues by one end only, as part of the chemical modification. The total molecular weight of the cross-linked dimer is approximately 116 kDa. Pegunigalsidase alfa has a specific activity of 35–62 U/mg. PEGylation is a proven strategy for increasing a drug’s half-life, enhancing pharmacodynamics, and reducing toxicity and immunogenicity. PEGylation improves the drug’s pharmacodynamics and multiplies its plasma half-life by ∼40-fold (about 80 h for pegunigalsidase alfa compared to ∼ ≤ 2 h for other currently available ERTs) (Kizhner et al., 2015).

The goal of PEGylation is typically to reduce renal clearance, block antibody and protein binding sites, increase the biological half-life, and thus boost effectiveness. The PEG moiety tends to be larger than the biologic (Turecek et al., 2016; Chen et al., 2021). In contrast, a relatively small bifunctional PEG linker of ∼2 kDa was optimal for pegunigalsidase alfa (Ruderfer et al., 2018). Nevertheless, PEGylation appears to have the expected beneficial effects on the enzyme’s properties; in preclinical studies *in vitro* and *in vivo*, pegunigalsidase alfa was more heat-stable and showed a longer plasma half-life (about 40-fold), greater enzyme activity, greater biodistribution, less liver clearance, and reduced neutralizing antibody (nAb) binding (Lenders et al., 2022; Lenders et al., 2023). PEGylation may also carry a theoretical potential for reduced immunogenicity due to epitope masking as suggested by *in vitro* data (Lenders et al., 2022) that warrants further immunology studies on real-world data.

Pegunigalsidase alfa (Elfabrio^®^, Chiesi Farmaceutici) is a novel chemically modified recombinant human α-Galactosidase ERT approved by the European Medicines Agency (EMA) and US Food and Drug Administration (FDA) for the treatment of Fabry disease. Pegunigalsidase alfa has been developed clinically in a number of settings (Table 1). A 12-month Phase 1/2 trial in patients with a relatively mild classic FD phenotype comprised a 3-month dose-ranging part (NCT01678898) and then a 9-month extension (NCT01769001) (Schiffmann et al., 2019). Sixteen patients completed the 12-month treatment period and displayed a stable mean eGFR and an apparent attenuated immune response. Although three patients initially developed ADAs, all were negative by the end of the clinical trial. Most treatment-emergent adverse events (TEAEs) were mild or moderate (Schiffmann et al., 2019). This small trial in ERT naïve patients was designed to prove the concept of effective tissue clearance by pegunigalsidase alfa by renal biopsies performed at baseline and after 6 months of treatment. The peritubular capillary Gb_3_ inclusions evaluated by the BLISS score (Barisoni Lipid Inclusion Scoring System) were reduced by 84% (Schiffmann et al., 2019).

**TABLE 1:** Clinical development program of pegunigalsidase alfa.

	Study number	Study name	NCT Clinicaltrial.gov number	Sponsor	Phase	Number of patients	Duration (months)	Dose of pegunigalsidase alfa	Reference
Dose-ranging study of PRX-102 in adult Fabry disease patients	PB-102-F01 and F02		NCT01678898 and NCT01769001	Protalix Biotherapeutics	Phase 1/2	19	3 + 9	0.2 mg/kg/2-week; 1 mg/kg/2-week; 2 mg/kg/2-week	Schiffmann R et al. J Inher Metab Dis 2018
A multi center extension study of PRX-102 administered by intravenous infusions every 2 Weeks for up to 60 Months to adult fabry patients	PB-102-F03		NCT01981720	Protalix Biotherapeutics	Phase 1/2 (extension study)	15	60 (12 + 48)	1 mg/kg/2-week	Hughes D et al. Genet Med 2023
Phase 3 open-label switch over study to assess safety, efficacy and PK of pegunigalsidase alfa (PRX-102) 2 mg/kg IV every 4 Weeks for 52 Weeks in Fabry disease patients currently treated with enzyme replacement therapy Fabrazyme^®^ or Replagal™	PB-102-F50	BRIGHT	NCT03180840	Protalix Biotherapeutics	Phase 3	30	12	2 mg/kg/4-week	Bernat J et al. Genet Med 2023 Suppl
An open label study of the safety and efficacy of PRX-102 in patients with Fabry disease currently treated with REPLAGAL^®^ (agalsidase alfa)	PB-102-F30	BRIDGE	NCT03018730	Protalix Biotherapeutics	Phase 3	22	12	1 mg/kg/2-week	Linhart A et al. Orphanet J Rare Dis 2023
A randomized, double blind, active control study of the safety and efficacy of PRX-102 compared to agalsidase beta on renal function in patients with Fabry disease previously treated with agalsidase beta	PB-102-F20	BALANCE	NCT02795676	Protalix Biotherapeutics	Phase 3	78	24	1 mg/kg/2-week	Wallace E et al. J Med Genet 2023
Open label extension study to evaluate the long-term safety and efficacy of pegunigalsidase alfa (PRX-102) in patients with Fabry disease	CLI-06657AA1-04 (formely PB-102-F60)	BRILLIANCE	NCT03566017	Chiesi Farmaceutici	Phase 3 (Open label extension study)	97	60	1 mg/kg/2-week	Unpublished

Fifteen ERT-naive adults with FD (8 males; 7 females) out of sixteen who completed the first 12 months of pegunigalsidase alfa treatment enrolled in a 60-month open-label extension study of 1 mg/kg pegunigalsidase alfa infusions every other week (Study F03, NCT01981720). Five of these patients discontinued prematurely. Ten completed ≥48 months (60 months total treatment) and enrolled in a further extension study (Study F60, NCT 03566017) (Hughes et al., 2023). Most treatment-emergent adverse events were mild/moderate in severity and all infusion-related reactions were mild/moderate in severity. Four patients were transiently positive for anti-pegunigalsidase alfa IgG. However, during the first infusion one patient developped bronchospasm resolving with epinephrine and corticosteroid treatment and was shown to have pre-existing IgE antibodies against pegunigalsidase alfa (Schiffmann et al., 2019).

Patients showed continuous reduction in plasma lyso-Gb_3_ levels with mean (standard error) reduction of 76.1 (25.1) ng/mL from baseline to month 24. After 60 months of treatment, the calculated mean (SE; median) annualized eGFR slope were −1.6 mL/min/1.73 m^2^/y for all patients, −2.4 mL/min/1.73 m^2^/y for male and −0.7 mL/min/1.73 m^2^/y for females. At month 60, mean left ventricular mass index (LVMi) had increased in females by 13.6 g/m^2^ (5.3) compared with 5.7 in males; no cardiac fibrosis was observed (Hughes et al., 2023).

The phase 3 clinical development program for pegunigalsidase alfa included three separate trials named “BRIDGE”, “BRIGHT”, and “BALANCE”, all of which have completed.

### The BRIDGE study (NCT03018730)

The BRIDGE study (completed in December 2019) was a Phase 3, open-label switch-over study of the safety and efficacy of pegunigalsidase alfa at the dose of 1 mg/kg every other week in adult patients with Fabry disease having previously been treated with agalsidase alfa for two or more years and a stable dose of the latter for at least the last 6 months (Linhart et al., 2023). Twenty-two patients completed the 12-month treatment period. The investigators reported a substantial improvement in the mean annualized eGFR slope in both male and female patients (from −5.90 mL/min/1.73 m^2^/year to −1.19 mL/min/1.73 m^2^/year on agalsidase alfa and pegunigalsidase alfa, respectively. However, the investigators pointed out that the pre-switch period of agalsidase alfa treatment was not closely controlled and that the clinical measurements were not standardized. Two patients (9.1%) withdrew due to a type I hypersensitivity reaction and were shown to be positive for IgE antibodies against the enzyme. The most common TEAEs (nasopharyngitis, headache and dyspnoea) were moderate in intensity (Linhart et al., 2023).

### The BRIGHT study (NCT03180840)

The BRIGHT study (completed in June 2020) was a multicentre, multinational, open-label, study of the safety, efficacy and pharmacokinetics of a switch to 52 weeks of pegunigalsidase alfa treatment in adult patients having received agalsidase beta or agalsidase alfa for at least 3 years. The pegunigalsidase alfa administration regimen was unusual in that the dose was 2 mg/kg every 4 weeks and not the 1 mg/kg every 2 weeks used in other trials. Thirty adult patients (24 males and 6 females; mean (standard deviation) age: 40.5 (11.3) years; age range: 19–58 years) were enrolled, 29 of whom completed the 1-year treatment period. The plasma lyso-Gb_3_ levels were stable throughout the treatment period. The overall mean (standard error) eGFR slope at the end of the study was −2.92 (1.05) mL/min/1.73 m^2^/year. None of the initially ADA-negative patients became positive during the treatment period. A total of 183 TEAEs were reported in nine (30.0%) of the patients; 33 were considered to be treatment related (infusion reactions, diarrhoea, erythema, fatigue, influenza-like illness, an elevated urine protein/creatinine ratio, and pyuria) (Bernat et al., 2023).

### The BALANCE study (NCT02795676)

The final results of the 2-year Phase 3 BALANCE study (PB-102-F20, NCT02795676) have recently been published (Wallace et al., 2023). The study was designed to assess the safety and efficacy of pegunigalsidase alfa in a head-to-head comparison with agalsidase beta in FD patients with declining renal function (Supplementary Figure S1).

The BALANCE study was a randomized, double-blind, head-to-head Phase 3 noninferiority study comparing pegunigalsidase alfa and agalsidase beta in FD patients with deteriorating renal function and previously treated with agalsidase beta for more than 1 year. The study was conducted at 29 centres in 12 countries in northern America and Europe, with recruitment between 2016 and 2021. The main inclusion criteria for males or females were age 18–60, at least one classical diagnostic feature of FD, an eGFR (calculated according to the CKD EPI Collaboration equation) of 40–120 mL/min/1.73 m^2^, and deteriorating kidney function (a linear eGFR slope more negative than -2 mL/min/1.73 m^2^/year over 9–18 months based on at least 3 serum creatinine measurements). The participants were randomized 2:1 to treatment with pegunigalsidase alfa or agalsidase beta (1.0 mg/kg every 2 weeks, for both) for 2 years.

### Primary and secondary endpoints

Trial PB-102-F20 was submitted in 2016 by Protalix Biotherapeutics for special protocol assessment by the FDA designed as a non-inferiority trial. The FDA did not agree with the design and recommended a superiority design due to the lack of data to support the proposed non-inferiority margin of −3 mL/min/1.73 m^2^/year/. Following FDA’s recommendation, the final protocol submitted in 2017 stated that the trial primary objective was to demonstrate superiority of pegunigalsidase alfa compared to agalsidase beta. However, while trial PB-102-F20 was ongoing, agalsidase beta received full approval from the FDA on 11 March 2021 - the full approval was supported by a phase 3 trial, a phase 4 trial, and a long-term observational study - (Food and Drug Administration (FDA), 2023). After the approval of agalsidase beta and discussions with the FDA, the primary objective of Trial PB-102-F20 (BALANCE) was changed during the course of the study from superiority to noninferiority of pegunigalsidase alfa compared with agalsidase beta at 24 months for regulatory submissions to the European Medicines Agency (EMA) and Food and Drug Administration (FDA), respectively (Wallace et al., 2023). To determine noninferiority, the lower boundary of a 95% confidence interval (CI) for the difference in the primary efficacy endpoint (the median annualized eGFR slope) was prespecified as −3 mL/min/1.73 m^2^/year (Wallace et al., 2023). Although the FDA agreed to the non-inferiority analysis in principle, no agreement was reached regarding Protalix’ proposed non-inferiority margin of −3 mL/min/1.73 m^2^/year and the FDA stated that based on the best available data comparing agalsidase beta to placebo an acceptable statistical margin would have been 0.5–0.6 mL/min/1.73 m^2^/year) (Food and Drug Administration (FDA), 2023). In contrast to the FDA, the EMA did not accept the change of the end-point and adhered to the 12 months non-inferiority (European Medicines Agency, 2024a).

As a secondary efficacy endpoint, the plasma level of lyso-Gb_3_ was assayed at baseline, 1.5 months, every 3 months during year 1 of the study, and every 6 months during year 2. The presence or absence of circulating ADAs was measured using an ELISA. With regard to safety; treatment-emergent adverse events (TEAEs) were recorded and classified as definitely, probably, or possibly related to treatment.

### What did the BALANCE study show?

After randomization, 52 patients received pegunigalsidase alfa and 25 received agalsidase beta. The pegunigalsidase alfa and agalsidase beta groups did not differ significantly with regard to the baseline median eGFR value (respectively 73.5 mL/min/1.73 m^2^ and 74.9 mL/min/1.73 m^2^), and respectively 48 and 24 patients completed the 24-month course of double-blind treatment. There were more female patients in the pegunigalsidase alfa group (n = 23/52 *versus* 7/25 in the agalsidase beta treated group). In an intention-to-treat analysis of the eGFR decline over 2 years, the intergroup difference [95%CI] in the median slope was −0.36 mL/min/1.73 m^2^/year [-2.44; 1.73]. The confidence interval (CI) had a lower limit above the prespecified value of −3 mL/min/1.73 m^2^/year and included zero (indicating that the intergroup difference was not significant). Furthermore, subgroup analyses of males, females, nAb-positive patients and nAb-negative patients in the two treatment groups indicated that the eGFR slopes did not differ significantly.

However, EMA stated that “no final conclusion on non-inferiority over agalsidase beta as measured by the annualised eGFR can be retrieved from the BALANCE study given that the data for the primary endpoint comparison at month 12 was not on its own sufficiently informative due to the design and size of the trial. Nevertheless, the median eGFR slopes from baseline to month 24 of pegunigalsidase and the comparator agalsidase beta appeared close”. At month 12, the mean slopes for eGFR were −2.507 mL/min/1.73 m^2^/year for the pegunigalsidase alfa arm and −1.748 for the agalsidase beta arm (difference −0.749 [-3.026, 1.507]. At month 24, the median slopes for eGFR were −2.514 [−3.788; −1.240] mL/min/1.73 m^2^/year for the pegunigalsidase alfa arm and −2.155 [−3.805; −0.505] for the agalsidase beta arm (difference −0.359 [−2.444; 1.726]) (European Medicines Agency, 2024a).

At 24 months, median (range) plasma lyso-Gb_3_ change from baseline in males was 5.30 (−32.2–32.7) nM with pegunigalsidase alfa and −2.40 (−102.3 to 2.4) nM with agalsidase beta; (*p* = 0.0001); in females, the change was minimal: 0.10 (−4.0–5.8) nM with pegunigalsidase alfa and −0.30 (−0.7–0.9) nM with agalsidase beta (*p* = 0.54) (Wallace et al., 2023). The individual analysis revealed that the increase in lyso-Gb3 by more than 10 nM or 20% occurred in patients with higher proteinuria (UPCR ≥1 g/g) and positive anti-drug antibodies who were more represented in the pegunigalsidase alfa group. Of note, one patient in the agalsidase beta dropped his lyso-Gb3 values by more than 100 nM. This rises a question about the patient´s compliance before randomization although patients were supposed to take at least 80% of the dose prescribed (Wallace et al., 2023).

The proportions of patients experiencing infusion-related reactions (21% in the pegunigalsidase alfa group and 24% in the agalsidase beta group) were lower than the value of 67% given for clinical trial participants in agalsidase beta’s summary of product characteristics (https://www.ema.europa.eu/en/documents/product-information/fabrazyme-epar-product-information_en.pdf) in these populations previously treated by agalsidase beta for an average of 6 years.

The proportion of participants experiencing mild or moderate treatment-related TEAEs was similar in both groups. However, after adjustment for the time exposed to the therapeutic, the proportions of participants with mild or moderate treatment-related TEAEs were respectively 3.6-fold and 7.8-fold higher for agalsidase beta. Over the course of the study, the proportion of patients with neutralizing antibodies fell from 33% to 15% in the pegunigalsidase alfa group and from 28% to 26% in the agalsidase beta group (Wallace et al., 2023). Treatment-induced *de novo* ADAs were detected in three patients in the pegunigalsidase alfa group and three in the agalsidase beta group (Wallace et al., 2023). In the BALANCE trial, hypersensitivity reactions including anaphylaxis were reported in two pegunigalsidase-treated patients who experienced anaphylaxis during the initial infusion and were positive for anti-pegunigalsidase alfa-IgE antibodies (Wallace et al., 2023). Although the risk of pegunigalsidase alfa-related hypersensitivity may be increased in certain patients with pre-existing ADA from prior ERT, a similar event described as bronchospasm was noted in the Phase 1/2 trial in an ERT naïve patient. A case of membranoproliferative glomerulonephritis with immune depositions in the kidney was reported during the BALANCE clinical trial (Wallace et al., 2023). This event led to a decline in renal function that slowly improved upon discontinuation of pegunigalsidase alfa but did not return to baseline by the end of the trial (Wallace et al., 2023). No data was presented with respect to possible PEG-related ADA (Chen et al., 2021; Lenders et al., 2023).

## Discussion

The results of the Phase 3 BALANCE study confirmed that pegunigalsidase alfa (Elfabrio^®^, Chiesi Farmaceutici) is an effective, treatment option with favourable safety profile in patients with classical FD, alongside agalsidase alfa (Replagal^®^, Takeda) (Schiffmann et al., 2001), agalsidase beta (Fabrazyme^®^, Sanofi) (Eng et al., 2001; Wanner et al., 2023), and the orally administered pharmacologic chaperone migalastat (Galafold^®^, Amicus Therapeutics) (Germain et al., 2016; Benjamin et al., 2017) (Figure 1). Pegunigalsidase alfa good tolerability was emphasized by the fact that 97% of the study participants who completed the BALANCE clinical trial decided to continue or start treatment with pegunigalsidase alfa in a 60-month, open-label extension study: CLI-06657AA1-04 (formerly PB-102-F60 or BRILLIANCE, NCT03566017) which objective is to evaluate the long-term safety, tolerability, and efficacy of 1 mg/kg pegunigalsidase alfa administered every other week in adult patients who have successfully completed studies PB-102-F03 (Hughes et al., 2023), PB-102-F20 (BALANCE) (Wallace et al., 2023) or PB-102-F30 (BRIDGE) (Linhart et al., 2023).

BALANCE was the first double-blind, randomized, controlled, head-to-head clinical trial of ERTs in adult patients with previous agalsidase beta treatment (the current gold-standard active treatment) and deteriorating renal function. In contrast, an earlier head-to-head comparison of agalsidase alfa and agalsidase beta in the Netherlands was not double-blind because the therapeutic’s quality and storage life after rebottling could not be guaranteed (Vedder et al., 2007). Furthermore, the BALANCE study used the approved dose of agalsidase beta (1.0 mg/kg every 2 weeks) rather than a lower dose (0.2 mg/kg every 2 weeks, aligned with that of agalsidase alfa) tested in a previous head-to-head trial (Vedder et al., 2007); this is important as the clinical effect appears to be dose-dependent (Tøndel et al., 2013; Kramer et al., 2018). Similar drawbacks exist for another randomized, open-label, registry-based trial - the Canadian Fabry Disease Initiative (CFDI; NCT 004551046) (Sirrs et al., 2014).

The BALANCE study had a number of limitations. Firstly, intergroup differences in ADA status and the occurrence of infusion-related reactions might have been underestimated because the patients had already been treated with agalsidase beta for a mean duration of 6 years (Wallace et al., 2023). Infusion-related reactions and neo-sensitization occur mostly in the first few years of ERT, and so the BALANCE participants were not in the most sensitive treatment period. Secondly, the ranges of baseline eGFR values were broad (30–126 in the pegunigalsidase alfa group, and 34–108 mL/min/1.73 m^2^ in the agalsidase beta group), which suggests that the study population was heterogenous with regard to renal damage. The selection of patients was based on pre-screening serum creatinine values and calculated eGFR slope. The sudden change in eGFR slope in the agalsidase beta arm from pre-trial median −7.8 mL/min/1.73 m^2^/year to −2.16 mL/min/1.73 m^2^/year may be explained by the poor reliability of historical eGFR data, possible regression to the mean phenomenon but also by administration of the exact dose per body weight. However, the methodological cause is highly probable and supported by the wide range of slopes calculated from historical data with added screening and baseline eGFR values which in some patients changed the slope to positive values (the range reported in pegunigalsidase alfa was up to +6.3 mL/min/1.73 m^2^/year).

Thirdly, the trial reported serological presence or absence of ADAs, rather than the exact titre. Hence, further investigations of ADA status and immunogenicity in patients treated with pegunigalsidase alfa are required (Lenders and Brand, 2022; Lenders et al., 2023). As an example, the risk of possible PEG emerging antibodies (especially in patients who received PEGylated mRNA vaccination for COVID-19 pandemic and may therefore be more sensitized) was not reported in the publication. However, it should be noted, that this does not preclude the potential advantage of pegylation in preventing immune responses. The affinity of antibodies towards pegunigalsidase alfa may be lower compared to the other enzymes possibly by pegylation masking some epitopes (Lenders et al., 2022; Lenders et al., 2023).

With respect to efficacy, the non-inferiority margin of −3 mL appears relatively high when considering Fabry disease natural history. Also, while renal function (assessed as the slope of eGFR calculated using the CKD-EPI formula) was extensively studied as the primary objective of the study, relatively few data were available for other body domain areas such as cardiac geometry and function which are of importance in patients with Fabry disease (Namdar, 2016; Linhart et al., 2020; Pieroni et al., 2021). This was, in part, due to the fact that the COVID-19 pandemic stroke during the conduct of the BALANCE clinical trial, thereby preventing patients to attend all scheduled visits.

The significant safety issues with pegunigalsidase alfa comprise type I hypersensitivity reactions in patients with pre-existing IgE antibodies. Although relatively rare, it mandates treatment initiation in hospital settings equipped to handle such a complication. The described case of immune-complex mediated glomerulonephritis represents another potential issue which may have been overlooked with either enzyme therapy (Debiec et al., 2014), as the rapid decline in eGFR and proteinuria may be attributed to rapid progression of Fabry disease itself would have been missed in the absence of renal biopsy.

## Conclusion

In summary, the results of the Phase 3 BALANCE trial showed that pegunigalsidase alfa is an effective, safe treatment option for patients with FD which adds to the specific therapeutic arsenal against FD. This option might be of particular relevance when remembering the worldwide shortage in agalsidase beta which occurred between year 2009 and 2011 (Linthorst et al., 2011; Smid et al., 2011; Lin et al., 2014; Pisani et al., 2017; Kramer et al., 2018) and for patients in whom the *GLA* variant is not amenable to chaperone therapy (Benjamin et al., 2017) or ERT with agalsidase alfa or agalsidase is poorly tolerated or poorly effective.
