# Supplementary figures and images for: Pegunigalsidase alfa: a novel, pegylated recombinant alpha-galactosidase enzyme for the treatment of Fabry disease
Source: Front Genet. 2024 Apr 12;15:1395287. doi: 10.3389/fgene.2024.1395287 (PMC11045972; doi:10.3389/fgene.2024.1395287)

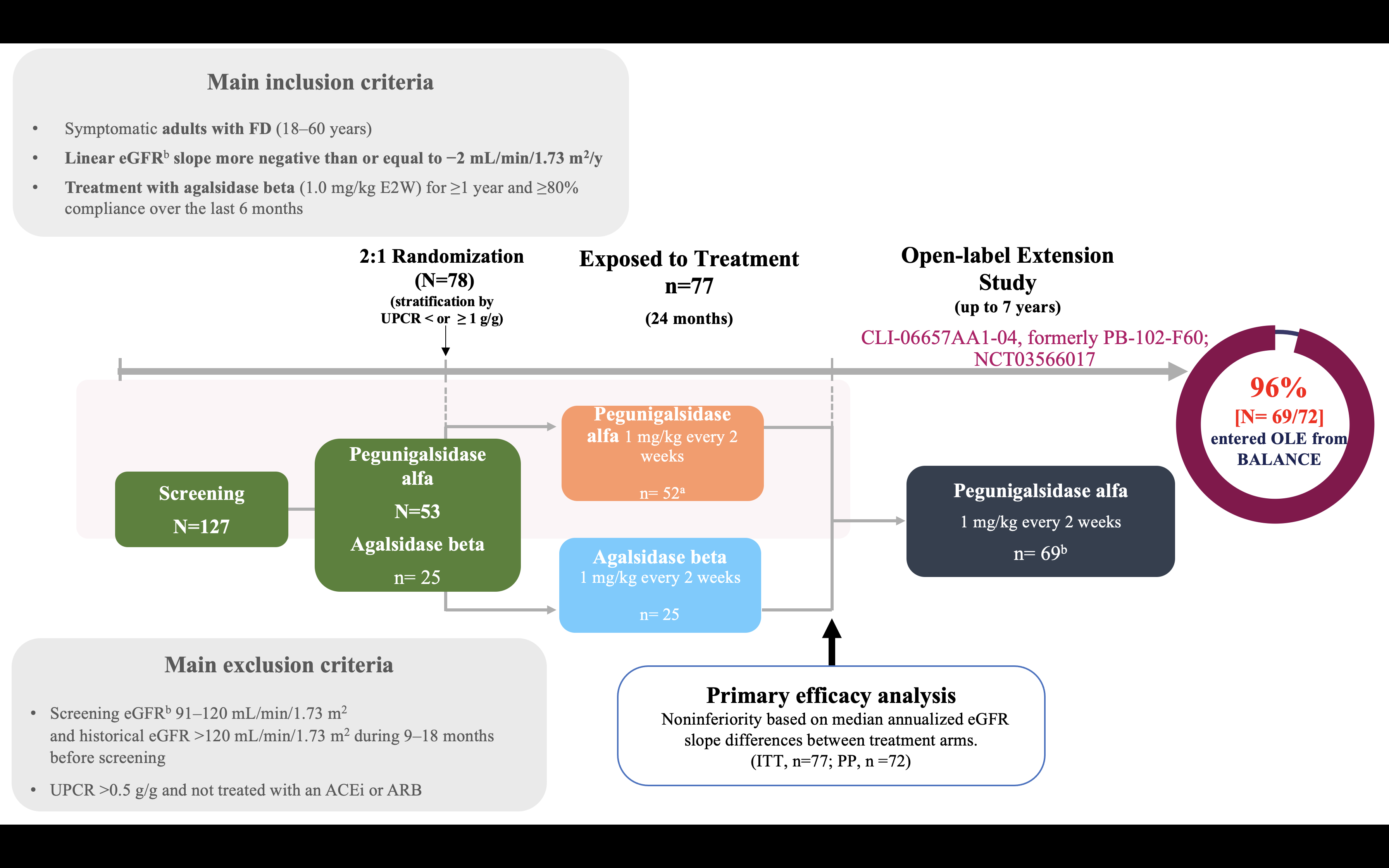

Supplement: Supplementary file 1 [file Image1.JPEG]
